# Evaluation of hematologic and coagulation changes associated with hyperfibrinolysis in feline acute trauma

**DOI:** 10.5455/javar.2026.m1030

**Published:** 2026-03-24

**Authors:** Nuriza Zamirbekova Erdoğan, Abdulkadir Önal, Merve İder, Kurtuluş Parlak, Furkan Çağrı Beşoluk

**Affiliations:** 1 Department of Surgery, Faculty of Veterinary Medicine, Selcuk University, Konya 63200, Türkiye; 2 Department of Internal Medicine, Faculty of Veterinary Medicine, Selcuk University, Konya 63200, Türkiye; 3 Department of Biostatistics, Faculty of Veterinary Medicine, Selcuk University, Konya 63200, Türkiye

**Keywords:** Acute traumatic coagulopathy, cat, hyperfibrinolysis, D-dimer

## Abstract

**Objectives:** This study investigates the hypothesis that hyperfibrinolysis may occur in cats with acute trauma, leading to alterations in hematological, biochemical, and coagulation parameters, particularly in severe cases. The aim is to assess these parameters (aPTT, PT, fibrinogen, and D-dimer) in traumatized cats and to examine their association with trauma severity and prognosis.

**Materials and Methods:** This study involved 35 acutely traumatized and 11 healthy cats. Trauma severity was quantified using the ATT (Multiple Trauma Triage Scoring System). For hematological, biochemical, and coagulation analyses, 2 ml of blood was drawn from a venous vein and processed within 15 to 30 min of collection.

**Results:** Traumatized cats exhibited significantly elevated CPK and WBC counts, alongside significantly reduced HCT and RBC counts (*p* < 0.05). Fibrinogen concentrations were significantly decreased. D-dimer levels and ATT scores were significantly increased in the trauma group (*p* < 0.05). No significant differences were observed between groups for PT, aPTT, TT, platelet count, or lactate levels (*p* > 0.05). Non-survivors had significantly higher lactate levels and lower pH, base excess (BE), and body temperature (*p* < 0.05).

**Conclusions:** This study demonstrated that ATT, lactate, pH, BE, and body temperature are effective predictors of both trauma severity and clinical prognosis in cats with acute trauma. Fibrin degradation products formed during this process are thought to contribute to elevated D-dimer levels, indicating the development of hyperfibrinolysis.

## 1. Introduction

ATC is a term used to describe the range of changes to the clotting process that occur following severe injury [1]. Coagulation disorders, also known as trauma-induced coagulopathy (TIC), affect around 28% of trauma patients and can lead to a four- to six-fold increase in mortality [2, 3]. Traumatic coagulopathy may develop due to multiple factors, including tissue factor–mediated activation of coagulation factors, hemodilution caused by resuscitation fluids, platelet dysfunction, endothelial dysfunction, activation of protein C, consumption and modification of fibrinogen, and hyperfibrinolysis [4]. Hyperfibrinolytic disorders are characterized by dysregulated fibrinolysis, which results in the premature breakdown of blood clots and bleeding [5, 6, 7]. Hyperfibrinolysis is initiated when the endothelium releases tissue plasminogen activator (tPA) into the bloodstream, leading to the degradation of fibrinogen and fibrin. The breakdown of fibrin produces soluble fibrin degradation products, including D-dimers.

Hemostatic disorders have been reported to develop following trauma in both cats and dogs [8, 9]. However, data on traumatic coagulopathic changes in cats are limited. Only a few studies support the presence of ATC, which is characterized by hypocoagulability and hyperfibrinolysis. Previous studies have primarily focused on investigating hemostatic abnormalities in traumatized dogs [8, 10].

This study was designed based on the hypothesis that hyperfibrinolysis may develop in cats with acute trauma and that it may cause changes in hematological, biochemical, and coagulation parameters. It is also hypothesized that these changes may be more pronounced in severe cases. This study aims to evaluate hematological, biochemical, and coagulation parameters (aPTT, PT, fibrinogen, and D-dimer) in cats exposed to trauma and to determine the relationship between these variables, trauma severity, and prognosis.

## 2. Materials and Methods

### 2.1. Ethical approval

All procedures were approved by the Ethics Committee of the Experimental Animal Production and Research Centre (SÜDAMEK) at the Faculty of Veterinary Medicine, Selçuk University, under reference number 2024/03. The owners of all cats included in the study were provided with detailed information about the research protocol and gave informed consent.

### 2.2. Study group

This study included 35 cats presenting to the Selçuk University Faculty of Veterinary Medicine Animal Hospital with acute trauma between 2024 and 2025. Trauma cases involved blunt injuries (high falls, vehicle accidents, etc.) occurring within the previous 12 h. Cats that had received intravenous fluids, corticosteroids, or anticoagulants were excluded. A control group of 11 clinically healthy cats of varying breeds, sexes, and ages was selected from patients visiting for routine check-ups or vaccinations. All cats underwent physical examinations, trauma scoring, and laboratory evaluations, including complete blood counts, venous blood gas analysis, and biochemical analyses. Control cats were confirmed clinically normal based on these assessments.

### 2.3. Clinical examination and scoring systems

The breed, age, and gender of the cats included in the study were recorded, and routine clinical examinations were performed. The ATT scoring system was used to assess the severity of trauma in traumatized cats. The ATT scoring system evaluated six categories: perfusion, cardiac, respiratory, eye/muscle/skin, skeletal, and neurological. Each category was scored on a scale of 0 (normal) to 3 (severe impairment) based on the extent of damage to the relevant system, yielding a total score of 0 to 18. The total ATT score was classified as ‘mild’ for cats with a score of 6 or less, ‘moderate’ for scores between 7 and 11, and ‘severe trauma’ for scores of 12 or higher [11, 12].

### 2.4. Blood sample collection and analysis

Blood samples were obtained via percutaneous puncture of the external jugular vein using a 20-gauge needle and a non-anticoagulant syringe. The collected samples were then promptly transferred into tubes designated for measurement. A total of 2 ml of blood was collected, of which 0.5 ml was transferred to K3-EDTA tubes for complete blood count analysis. This includes the following: white blood cells (WBCs), lymphocytes, monocytes, granulocytes, hematocrit (HCT), hemoglobin (Hb), red blood cells (RBCs), platelets (PLTs), mean corpuscular volume (MCV), mean corpuscular hemoglobin (MCH), and mean corpuscular hemoglobin concentration (MCHC). The complete blood count was performed using a Mindray BC-5000 Vet Automated Hematology Analyzer. Additionally, lactate concentrations and base excess (BE) levels were measured using a Siemens RAPID Point 500 blood gas analyzer from 0.3–0.4 ml of venous blood transferred to heparinized tubes. 0.8 ml of blood was transferred to tubes without an anticoagulant, left to clot at room temperature for 15 min, and then centrifuged at 5,000 x *g* for 10 min to obtain the serum. Serum CPK levels were measured using a BT 3000 Plus analyzer on a portion of the obtained serum.

For the D-dimer analysis, 0.5 ml of venous blood was collected in a 1.5 ml Eppendorf tube containing 50 µl of sodium citrate. The samples were left to stand at room temperature for 15 min, after which they were centrifuged at 5,000 x *g* for 5 min. The resulting plasma was analyzed using a Vcheck V200 Automatic Veterinary Hormone and Immunoassay Analyzer. According to the analysis, values below 0.3 µg/ml were considered normal, while those above 0.3 µg/ml were considered significant for the study and outside the reference range. Meanwhile, PT, aPTT, thrombin time (TT), and fibrinogen parameters were analyzed in the remaining plasma samples using a coagulometer (Sysmex CA-560, Siemens, Germany).

### 2.5. Statistical analysis

Statistical analysis was performed to determine parameters associated with traumatized cats. Data were tested for normality using the Shapiro-Wilk test. When comparing two group means, normally distributed data were reported as mean ± standard deviation and analyzed using the independent samples t-test. Non-normally distributed data were presented as median (min-max) and analyzed using the Mann-Whitney U test. A *p*-value less than 0.05 was considered statistically significant. Differences in blood parameters between the trauma and control groups were assessed using the t-test and Mann-Whitney U test, and correlations were assessed using the Spearman rank correlation coefficient. Statistical analyses were performed using IBM SPSS Statistics for Windows v27.0 (IBM Corp., Armonk, NY).

## 3. Results

### 3.1. Clinical examination findings of healthy and acutely traumatized cats

A total of 11 healthy cats (6 males, 54.55%, and 5 females, 45.45%) and 17 females (48.57%) and 18 males (51.43%) with acute trauma participated in the study. The breed distribution of the healthy cats was as follows: Six domestic shorthair mixed-breed cats (54.54%), two Scottish Fold cats (18.18%), one British Shorthair cat (9.09%), and one British Longhair cat (9.09%). The group of cats with acute trauma comprised 21 domestic shorthair mixed-breed cats (60%), seven British shorthairs (20%), three Turkish Angoras (8.57%), two Bombays (5.71%), one Siamese (2.86%), and one Scottish fold (2.86%). The mean age of healthy cats was 13 months (range 7–24 months), while the mean age of acutely traumatized cats was 16 months (range 2–54 months). Traffic accidents were identified as the cause of trauma in 57.14% of the acutely traumatized cats (*n* = 20), falls from height in 40% (*n* = 14), and dog bite injuries in 2.86% (*n* = 1).

A thoracic injury was identified in 62.86% (*n* = 22) of acutely traumatized cats. Of these, pulmonary contusion was detected in 10 cases, pneumothorax in 7, and hemothorax in 5. Additionally, cranial trauma was observed in 11 cases, anisocoria in three cases, lethargy in three cases, stupor in six cases, and paraplegia in three cases. Injuries to the musculoskeletal system were characterized by multiple fractures in 12 cases, single fractures and soft tissue injuries to the extremities in seven cases, open fractures and multiple fractures in six cases, soft tissue injuries only in seven cases, and a combination of fractures, soft tissue injuries, and an abdominal hernia in two cases. Among the acutely traumatized cats, hypothermia was recorded in five cases (14.29%), normothermia in 21 cases (60%), and hyperthermia in nine cases (25.71%). The survival rate was 71.43% (25 cats), while 10 (28.57%) died.

### 3.2. Blood gas, hemogram, and biochemical analysis findings

CPK (*p* = 0.000) and WBC (*p* = 0.016) levels were found to be significantly higher in acutely traumatized cats than in healthy controls, while HCT (*p* = 0.012) and RBC (*p* = 0.012) counts were significantly lower (Table 1). No significant differences were observed in platelet counts or lactate levels between healthy cats and acutely traumatized cats (*p* > 0.05).

**Table 1.** Comparison of venous blood gas, biochemistry, and complete blood count parameters between the control group and cats with acute trauma.

Parameters	Groups	Reference Range	Mean ± SD	*p*-value
Lactate (mmol/l)	Control group	0.6–2.2	2.36 ± 0.97	0.085
	Acute trauma		3.60 ± 2.25	
CPK (U/l)	Control group	69–214	469.82 ± 265.16	0.000*
	Acute trauma		10939.60 ± 12509.56	
WBC (10^9^ /l)	Control group	7.35–7.45	8.71 ± 1.95	0.016*
	Acute trauma		11.56 ± 5.76	
HCT (%)	Control group	0.26–0.47	37.91 ± 3.72	0.012*
	Acute trauma		31.27 ± 8.04	
RBC (10^12^ /l)	Control group	4.6–10.2	10.78 ± 1.00	0.000*
	Acute trauma		7.81 ± 2.11	
Platelets (10^9^ /l)	Control group	100–518	141.73 ± 80.55	0.139
	Acute trauma		187.34 ± 89.52	

*: *p* < 0.05. Abbreviations: CPK, creatine phosphokinase; WBC, white blood cells; HCT, hematocrit; RBC, red blood cells. Note: The independent samples *t*-test was used for parameters with normal distribution.

### 3.3. Coagulation parameters and ATT results

**Figure 1.**
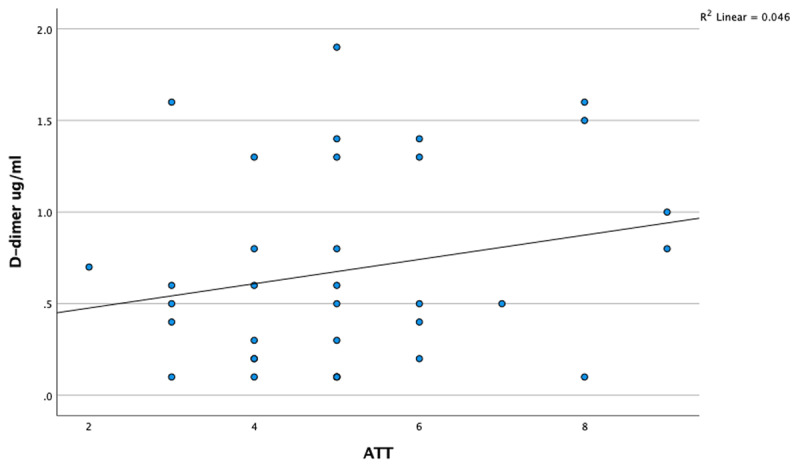
Changes in D-dimer levels in relation to the ATT score in acutely traumatized cats.

**Figure 2.**
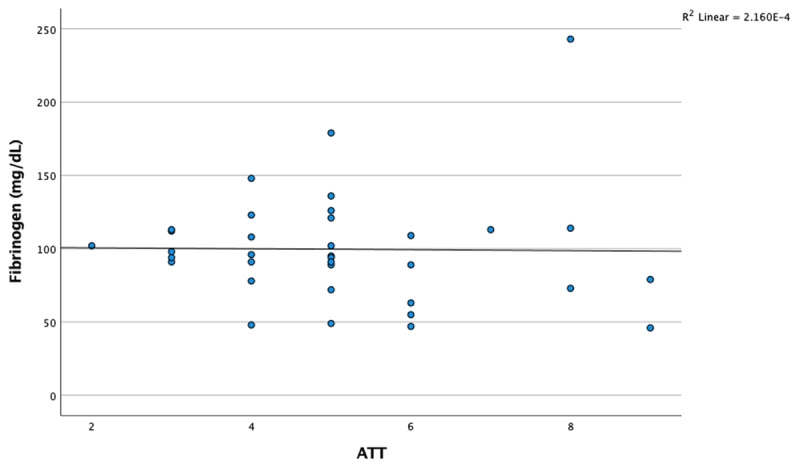
Changes in fibrinogen levels in relation to the ATT score in acutely traumatized cats.

Fibrinogen levels in cats with acute trauma were significantly lower (*p* = 0.036). The D-dimer level (*p* = 0.000) and ATT score were significantly higher (*p* = 0.000) than in healthy cats. There were no significant differences in the PT, aPTT, and thrombin parameters between cats with acute trauma and healthy cats (*p* > 0.05) (Table 2). Between D-dimer levels and ATT scores, there was a weak positive correlation, but this relationship was not statistically significant, ρ(35) = 0.176, *p* = 0.311, 95% CI [–0.177, 0.487]. On the other hand, between fibrinogen levels and ATT scores, there was a weak negative correlation, but this relationship was also not statistically significant, ρ(35) = –0.199, *p* = 0.252, 95% CI [–0.507, 0.154] (Figures 1, 2).

**Table 2.** Comparison of coagulation parameters and ATT scores between the control group and cats with acute trauma.

Parameters	Groups	Reference Range	Mean ± SD or Median (Min–Max)	*p*-value
ATT	Control group		0.00 (0–1)	0.000*
	Acute trauma		5.00 (2–9)	
aPTT (sec)	Control group	15.0–21.0	40.28 ± 24.23	0.745
	Acute trauma		37.89 ± 20.21	
Trombin (ng/ml)	Control group	75–100	158.27 ± 67.51	0.965
	Acute trauma		159.51 ± 84.09	
PT (sec)	Control group	15.0–20.0	12.40 (8.90–49.60)	0.800
	Acute trauma		11.60 (8.70–26.40)	
Fibrinogen (mg/dl)	Control group	150–300	113.00 (76–263)	0.036*
	Acute trauma		95.00 (46–243)	
D-dimer ug/ml	Control group	Normal > 0.3 > Abnormal	0.10 (0.10–0.20)	0.000*
	Acute trauma		0.50 (0.10–1.90)	

*: *p* < 0.05. Abbreviations: ATT, Animal Trauma Triage score; aPTT, activated partial thromboplastin time; PT, prothrombin time. Note: An independent samples *t*-test was used for normally distributed variables (aPTT, thrombin), while the Mann–Whitney U test was applied for non-normally distributed variables (ATT, PT, fibrinogen, D-dimer).

### 3.4. Mortality findings

The lactate levels were found to be significantly higher (*p* = 0.027), while the pH (*p* = 0.001), BE, and body temperature were found to be significantly lower (*p* = 0.000). There were no significant differences in WBC, RBC, HCT, CPK, PT, aPTT, fibrinogen, thrombin, or ATT score levels between survivors and non-survivors with acute trauma (*p* > 0.05) (Table 3).

**Table 3.** Comparison of venous blood gas parameters, complete blood count, and coagulation values between surviving and non-surviving cats with acute trauma.

Parameters	Groups	Reference Range	Mean ± SD or Median (Min–Max)	*p*-value
Platelets	Survived	100–518	178.76 ± 74.06	0.378
	Died		208.80 ± 122.20	
WBC (10^9^/l)	Survived	5.5–19.5	12.12 ± 5.26	0.370
	Died		10.16 ± 6.95	
RBC (10^12^/l)	Survived	4.6–10.2	7.83 ± 2.16	0.923
	Died		7.76 ± 2.11	
PT (sec)	Survived	15.0–20.0	13.90 ± 5.40	0.521
	Died		15.22 ± 5.49	
aPTT (sec)	Survived	15.0–21.0	37.04 ± 20.55	0.699
	Died		40.02 ± 20.25	
Fibrinogen (mg/dl)	Survived	150–300	97.24 ± 29.94	0.569
	Died		105.60 ± 56.00	
D-dimer (μg/ml)	Survived	Normal > 0.3 > Abnormal	0.68 ± 0.54	0.961
	Died		0.69 ± 0.55	
ATT	Survived		4.68 ± 1.41	0.056
	Died		6.20 ± 2.10	
pH	Survived	7.35–7.45	7.33 ± 0.06	0.001*
	Died		7.23 ± 0.09	
BE (mmol/l)	Survived	–5/+5	–8.68 ± 2.61	0.000*
	Died		–13.05 ± 3.44	
Temp (°C)	Survived	36.7–38.9°C	38.17 ± 0.80	0.000*
	Died		36.76 ± 1.23	
Lactate (mmol/l)	Survived	0.6–2.2	2.85 ± 1.22	0.027*
	Died		5.48 ± 3.12	
CPK (U/l)	Survived	69–214	5425 (202–36124)	0.928
	Died		7191.50 (24–37892)	
HCT (%)	Survived	0.26–0.47	31.00 (4.90–42.30)	0.788
	Died		31.85 (24–55.30)	
Trombin (ng/ml)	Survived	75–100	146.00 (62–486)	0.339
	Died		129.50 (80–317)	

*: *p* < 0.05. Abbreviations: WBC, white blood cells; RBC, red blood cells; PT, prothrombin time; aPTT, activated partial thromboplastin time; ATT, Animal Trauma Triage score; BE, base excess; CPK, creatine phosphokinase; HCT, hematocrit. Note: An independent samples *t*-test was used for normally distributed parameters, while the Mann–Whitney U test was applied for non-normally distributed data (CPK, HCT, thrombin).

In acute traumatic deaths, pH, BE, body temperature, and lactate levels were evaluated for their ability to predict mortality. Both pH and BE showed 76% sensitivity and 78% specificity. Body temperature showed the highest sensitivity (84%) and specificity (78%), while lactate levels showed a sensitivity of 77% and a specificity of 70% (Table 4).

**Table 4.** ROC analysis results: AUC, standard error, 95% confidence intervals, optimal cutoff, sensitivity, and specificity for predicting mortality in cats.

Variable	AUC	Standard error	*p*-value	Asymptotic 95% confidence interval		Sensitivity (%)	Specificity (%)	Cut-Off value
				Lower Band	Upper Bound			
pH	0.776	0.098	0.015*	0.584	0.976	76	78	7.28
BE	0.816	0.071	0.005*	0.676	0.956	76	78	–10.85
Temp	0.831	0.101	0.003*	0.633	1.000	84	78	37.35
Lactate (mmol/l)	0.763	0.096	0.020*	0.574	0.951	77	70	3.40

*: *p* < 0.05.

## 4. Discussion

This study investigated the development of hyperfibrinolysis in cats by evaluating D-dimer, fibrinogen, PT, aPTT, thrombin, platelet count, lactate, and CPK levels, and examining their relationships with the ATT scoring system and prognosis. The results demonstrated that D-dimer, lactate, CPK, and ATT scores were significantly elevated in cats with acute trauma, whereas fibrinogen levels were significantly decreased. Furthermore, elevated lactate concentrations, high ATT scores, low pH values, reduced BE, and decreased body temperature were identified as important indicators of mortality in acutely traumatized cats. Collectively, these findings suggest that fibrinogen reserves are depleted due to activation of the fibrinolytic system in response to acute trauma. The resulting fibrin degradation products contribute to elevated D-dimer levels, indicative of hyperfibrinolysis.

The majority of traumatic injuries in cats are attributed to external causes, particularly vehicular accidents and falls from heights. Previous studies have reported traffic accidents as the cause of trauma in 56.2% and 59.3% of cases, respectively [13, 14]. Similarly, the present study found that traffic accidents accounted for 57.14% of acute trauma cases, aligning closely with these earlier findings. In contrast, trauma due to falls from heights was observed in 40% of cases—higher than the 24% reported by Lee et al. [9]. This discrepancy may be linked to a higher proportion of cat ownership in apartment buildings in the study area, increasing exposure to open windows and balconies. Most trauma cases involved young cats, with a mean age of 16 months, consistent with previous reports [15, 16]. No significant sex-based differences in trauma incidence were observed.

High-rise syndrome and traffic accidents are among the most common causes of trauma in veterinary medicine, frequently resulting in musculoskeletal and thoracic injuries. In the present study, thoracic injuries were identified in 62.86% of traumatized cats, consistent with previous reports [15, 17], with pulmonary contusions and pneumothorax present in 77.27% of these cases [15, 18]. Musculoskeletal injuries were also prevalent, affecting 71.43% of patients (*n* = 25), with multiple fractures noted in the majority (*n* = 16), in agreement with findings by Lefman and Prittie [17]. and Vnuk et al. [19]. Head trauma, particularly orofacial injuries, was observed in 20% of cases, which is lower than the 51.8% reported by Bonner et al. [20], potentially due to differences in sample size and study duration. Abdominal injuries such as hemoperitoneum, traumatic pancreatitis, pancreatic rupture, uroabdomen, abdominal hernias, and urinary bladder injuries have been described in the literature, with abdominal hernias and bladder ruptures being less common [19]. In line with these findings, only two cases of abdominal hernia were diagnosed in the current study [19].

Cats are particularly vulnerable to high morbidity and mortality rates resulting from traumatic injuries, which represent clinical emergencies with potentially severe outcomes. The ATT score is a system for classifying the severity of trauma in such cases. Each point increase in the ATT score indicates greater trauma severity and reduces the probability of survival by approximately 2.3–2.6 times [18, 21, 22]. Recent studies have highlighted the high predictive accuracy of the ATT score in determining mortality in traumatized cats and dogs [18, 21, 22]. For instance, Muri et al. [23] reported a high mortality rate among cats with an average ATT score of 6. These findings are consistent with our study’s results. Specifically, only six out of 35 traumatized cats had a total ATT score below 3, whereas the mean ATT score among the remaining 29 cats was 5.5. Among cats that succumbed to their injuries, the mean ATT score was 6.2. These individuals accounted for 28.57% of all cases. Based on these observations, the ATT scoring system appears to have significant clinical utility for grading trauma severity and as a prognostic indicator in cats with acute trauma. Furthermore, previously reported mortality rates in cats suffering from acute trauma have ranged from 11.9% to 33.74% [13, 14, 15]. The mortality rate observed in our study falls within this range. Additionally, the presence of multiple injuries emerged as a critical factor influencing mortality. In our cohort, injuries involving multiple anatomical regions were observed in 82.86% (*n* = 29) of cases. ATT scores were significantly higher in these cases. Notably, an increase in the ATT score, especially in the context of polytrauma, was found to significantly elevate the risk of mortality in cats with acute trauma.

Although reductions in HCT, Hb, and RBC levels following acute trauma are often attributed to blood loss, some studies suggest hemodilution as an alternative explanation [24, 25, 26]. To address these differing interpretations, a study involving 120 splenectomized dogs reported significant pre- and postoperative decreases in HCT and Hb levels, concluding that these changes were most likely due to hemorrhage [27]. In line with these findings, the current study proposes that the marked reductions in HCT and RBC concentrations in traumatized cats are most plausibly due to hemorrhage. Previous studies have shown that trauma, particularly polytrauma, can result in elevated serum CPK activity due to muscle damage and ischemia [28, 29]. Similarly, this study found increased serum CPK levels in cats with acute trauma, likely reflecting muscle injury and ischemic processes. Although CPK activity was higher in non-surviving cats, it did not demonstrate prognostic significance.

When tissue perfusion is compromised, the body transitions from oxidative to anaerobic metabolism at the cellular level. This metabolic shift results in the accumulation of lactate, subsequently leading to the development of metabolic acidosis. Metabolic acidosis is characterized by elevated lactate levels, accompanied by reduced blood pH and BE. Previous studies have demonstrated that a decrease in body temperature (below 37.8°C) and an increase in blood lactate concentrations are associated with the severity of shock and trauma in cats and dogs admitted to emergency clinics [30]. A negative correlation between these two parameters has also been observed [30]. Furthermore, hyperlactatemia (≥ 4.5 mmol/l) in conjunction with hypotension has been identified as a significant predictor of mortality in feline patients [31]. Gottlieb et al. [12] reported that increased plasma lactate concentrations were associated with acute traumatic cerebral injury (ATCI) in traumatized cats and that this elevation correlated with clinical parameters, including diminished mentation (MA) and decreased state of consciousness (G) scores. The same study also identified associations between low G scores and abnormalities in coagulation parameters, hypoperfusion, hypothermia, reduced BE, and decreased pH levels [12]. In the present study, blood lactate levels were significantly higher (mean 5.48 mmol/l), while pH (mean 7.23), BE (mean –13.05 mmol/l), and body temperature (mean 36.76°C) were significantly lower in cats that did not survive compared to those that did. Receiver Operating Characteristic (ROC) analysis revealed that the most effective parameters for predicting mortality were pH (76% sensitivity, 78% specificity), BE (76% sensitivity, 78% specificity), body temperature (84% sensitivity, 78% specificity), and lactate levels (77% sensitivity, 70% specificity). These findings indicate that hypoperfusion, hypoxia, and hyperlactatemia develop in cats with acute trauma in association with the severity of the injury and that these alterations play a significant role in predicting clinical outcomes. The results support previously established associations between hyperlactatemia and increased mortality [32], reaffirming that lactate concentration serves as a critical biomarker for prognostication in trauma cases. Furthermore, reductions in pH, BE, and body temperature are considered reflective of the systemic physiological impact of trauma. These parameters may serve as valuable prognostic indicators of systemic perfusion deficits and inadequate tissue oxygenation in feline patients suffering from acute trauma [32].

Following trauma, the body activates the coagulation process to stop bleeding while also triggering the anticoagulant and fibrinolytic pathways to prevent excessive clotting. Hypoperfusion and hypoxia cause endothelial cells to release thrombomodulin, which binds with thrombin to form the Tr–TM complex. This complex activates protein C, which inhibits factors V and VIII and reduces free thrombin. This limits fibrin formation. While this helps to prevent abnormal clotting, it also increases the risk of hyperfibrinolysis [33]. Post-traumatic hyperfibrinolysis is initiated by an elevation in tissue plasminogen activator (tPA) levels, which results from the inhibition of plasminogen activator inhibitor-1 (PAI-1) by activated protein C. This cascade leads to enhanced degradation of fibrin or fibrinogen by plasmin, resulting in increased concentrations of fibrin degradation products, including D-dimer, in the circulation [33]. Consequently, elevated D-dimer levels are regarded as an indirect marker of systemic fibrinolytic activity [34].

D-dimer is utilized to assess coagulopathic processes that arise following trauma, particularly in the evaluation of disseminated intravascular coagulation (DIC), in conjunction with fibrinogen, PT, and aPTT assays. Tholen et al. [34] reported that 67% of cats diagnosed with DIC had elevated D-dimer concentrations, with D-dimer demonstrating a specificity of 56%. Furthermore, the study by Stokol et al. [35] revealed improved sensitivity and specificity values when serum or plasma fibrin degradation products (FDP) and D-dimer measurements were combined. In this study, the finding that D-dimer levels exceeded the reference range in 68.57% of cats (24 out of 35) with acute trauma supports the notion that the fibrinolytic system is activated following acute trauma in cats and that hyperfibrinolysis mechanisms may play a role.

Fibrinogen, an essential component of the coagulation system and a liver-derived acute-phase protein, plays a critical role in hemostasis. In traumatic coagulopathies, plasma fibrinogen levels decrease due to increased consumption and fibrinolysis, and this reduction has been associated with increased mortality [36, 37]. ATC in cats and dogs is characterized by decreased fibrinogen levels, along with prolonged PT and aPTT [12]. In the present study, significantly lower fibrinogen concentrations in traumatized cats compared to the healthy control group suggest that increased fibrinogen consumption, most likely due to hyperfibrinolysis, contributed to the reduction in plasma fibrinogen levels in traumatized cats. In addition, the concomitant increase in D-dimer levels and decrease in fibrinogen concentrations in cats with acute trauma indicate that fibrinogen is consumed during the fibrinolytic process, leading to the generation of fibrin degradation products that may elevate D-dimer levels.

Previous studies have demonstrated an association between increased ATT scores, which assess trauma severity, and the severity of trauma-induced coagulopathic changes [23]. In the present study, no statistically significant correlation was observed between ATT scores and D-dimer or fibrinogen levels. Nevertheless, D-dimer concentrations tended to increase, and fibrinogen levels tended to decrease in correspondence with higher ATT scores (Figures 1, 2). Although these findings imply the absence of a direct relationship between ATT scores and coagulation parameters, the study’s limited sample size may have contributed to the lack of statistical significance.

Traditional tests, such as INR, PT, aPTT, PLT, and fibrinogen, only measure the initial stages of coagulation in the diagnosis of acute traumatic coagulopathy associated with hyperfibrinolysis. They do not assess important processes such as platelet function, fibrinolysis, thrombin generation, or interactions with cell surfaces. While these tests are useful for monitoring bleeding and guiding blood product use, they are limited in their ability to diagnose and manage acute traumatic coagulopathy (ATC). Park et al. [38] reported that PT and aPTT tests have insufficient sensitivity for diagnosing traumatic coagulopathy, do not provide information on clot durability or conversion rates, and may exhibit variability in response to small measurement errors [38]. The present study similarly found no statistically significant differences in PT, aPTT, or platelet counts between traumatized and healthy cats.

Additionally, D-dimer and fibrinogen levels, as well as plasma lactate concentrations, did not show a significant correlation with the ATT score, which reflects the severity of trauma. These findings suggest that traditional coagulation tests may be ineffective for detecting hyperfibrinolysis in cats with acute trauma, consistent with previous studies [12, 39].

### 4.1. Limitations

This study has some limitations. First, previous studies have shown that hyperfibrinolysis can occur in association with various underlying pathological conditions such as hemoperitoneum, breast cancer, abdominal neoplasia, and chronic liver failure [7]. Since the chronic disease history of the traumatized cats included in this study was not thoroughly investigated, it would not be appropriate to attribute the development of hyperfibrinolysis solely to trauma. Secondly, viscoelastic hemostatic tests such as TEG and ROTEM, which comprehensively and in real time evaluate the coagulation process, were not used in this study due to budget and equipment limitations. Contrary to expectations, no correlation was found between ATT and coagulation parameters in this study. As a result, the study’s findings were interpreted with caution, and more extensive, in-depth clinical studies with larger sample sizes are required.

## 5. Conclusions

In conclusion, ATT scores, along with lactate, pH, BE, and body temperature, are effective predictors of trauma severity and clinical prognosis in cats with acute trauma. The formation of fibrin degradation products during this process contributes to elevated D-dimer levels, indicating the development of hyperfibrinolysis. Additionally, decreased fibrinogen levels reflect the activation of the fibrinolytic system in response to acute injury, leading to fibrinogen consumption. These findings highlight the importance of monitoring these parameters to assess trauma severity and guide clinical management in affected cats.

## Data Availability

The data presented in this study are available from the corresponding author upon reasonable request.

## List of abbreviations

ATC, acute traumatic coagulopathy; aPTT, activated partial thromboplastin time; ATT, Animal Trauma Triage scoring system; BE, base excess; CPK, creatine phosphokinase; CBC, complete blood count; FİB, fibrinogen; HCT, hematocrit; Hb, hemoglobin; MCV, mean corpuscular volume; MCH, mean corpuscular hemoglobin; MCHC, mean corpuscular hemoglobin concentration; PT, prothrombin time; PLT, platelet count; RBC, red blood cells; SÜDAMEK, Ethics Committee of the Experimental Animal Production and Research Centre; TIC, Trauma-Induced Coagulopathy; WBC, white blood cells.
